# Disability Benefit Reform and Benefit Outcomes: A Re-examination of the Introduction of the UK Personal Independence Payment Using Linked Population Data

**DOI:** 10.23889/ijpds.v9i5.2604

**Published:** 2024-09-18

**Authors:** Ana-Corina Miller, Duncan McVicar, Neil Rowland, Babak Jahanshahi

**Affiliations:** 1 Queen's University Belfast

## Objectives

Many countries face the problem of how to provide support for additional costs faced by disabled people while restricting disability benefit program growth given tight public finances. In 2013, the UK reformed its main additional costs disability benefit with this objective, changing both medical screening and payment rates with the introduction of the Personal Independence Payment (PIP). This study uses newly available linked administrative population data to examine the impact of this major reform on disability benefit receipt.

## Approach

We use the newly available 2011CBI dataset, which links Census records to microdata on benefit receipt for the population of England and Wales from 2011-2015. Difference-in-difference and synthetic control methods are used to estimate the reform impact on the probability of flowing onto disability benefits, exploiting the structure of the roll-out of PIP’s introduction across English regions.

## Results

Introducing PIP led to a sharp fall in the probability of disability benefit onflow, with tentative evidence of a partial reversal in the following year. Further analysis is required to quantify the extent to which this reflects well-known administrative delays in processing applications as opposed to a step change in the probability of claims being successful.

## Conclusions

This study is the first to present linked administrative population data estimates of the benefit recipiency effects of the introduction of PIP. In doing so it both contributes to the wider international literature on disability benefit reform and provides an early research application of a significant new administrative dataset for England and Wales.

